# Metabolic, Microvascular, and Structural Predictors of Long-Term Functional Changes Evaluated by Multifocal Electroretinogram in Type 1 Diabetes

**DOI:** 10.3390/biomedicines12112614

**Published:** 2024-11-15

**Authors:** Mariacristina Parravano, Serena Fragiotta, Eliana Costanzo, Fabiana Picconi, Paola Giorno, Daniele De Geronimo, Daniela Giannini, Monica Varano, Vincenzo Parisi, Lucia Ziccardi

**Affiliations:** 1 IRCCS-Fondazione Bietti, 00198 Rome, Italy; eliana.costanzo@fondazionebietti.it (E.C.); paola.giorno@fondazionebietti.it (P.G.); daniele.degeronimo@fondazionebietti.it (D.D.G.); daniela.giannini@fondazionebietti.it (D.G.); monica.varano@fondazionebietti.it (M.V.); vincenzo.parisi@fondazionebietti.it (V.P.); lucia.ziccardi@fondazionebietti.it (L.Z.); 2 Departmental Faculty of Medicine, UniCamillus-Saint Camillus International University of Health Sciences, 00131 Rome, Italy; 3 Ophthalmology Unit, NESMOS Department, Faculty of Medicine and Psychology, St. Andrea Hospital, Sapienza University of Rome, 00189 Rome, Italy; serena.fragiotta@uniroma1.it; 4 Unit of Endocrinology, Diabetes and Metabolism, Fatebenefratelli Isola Tiberina Gemelli Isola, 00186 Rome, Italy; fabipicco@gmail.com

**Keywords:** type 1 diabetes mellitus, multifocal electroretinography (mfERG), optical coherence tomography (OCT), optical coherence tomography angiography (OCTA), diabetic retinopathy

## Abstract

Background: This study aimed to analyze the potential pathogenic connection between metabolic factors, photoreceptor cell rearrangements, retinal microvascular perfusion, and functional parameters through multifocal electroretinography (mfERG) in type 1 diabetes mellitus (DM1). Methods: This prospective observational cohort study enrolled DM1 patients (40.5 ± 9.1 years) with mild nonproliferative diabetic retinopathy followed for 4 years. Patients were subjected to multimodal imaging, which included color fundus photography, optical coherence tomography (OCT), OCT angiography, adaptive optics (AO), and mfERG. OCTA slabs were analyzed using ImageJ software (software version 2.3.0/1.53f) to calculate perfusion density (PD) at both superficial (SCP) and deep (DCP) capillary plexuses, as well as flow deficit percentage (FD%) at the choriocapillaris (CC). To calculate cone metrics on AO at the parafovea, including cone density (CD), linear dispersion index (LDi), and heterogeneity packing index (Hpi%) in the parafovea, the images were post-processed using a MATLAB algorithm. The mfERG P1 implicit time (IT) and N1-P1 response amplitude density (RAD) from R1 (foveal area), R2 (parafoveal area), and the unified rings R1 + R2 were evaluated. Results: A total of 22 patients (22 eyes) were enrolled. No significant differences were noted in central mfERG amplitude and implicit time-averaged values (*p* > 0.05, all). The main factor influencing R1 IT was HbA1c, while R1 RAD was affected by Hpi and CC FD%. R1 + R2 IT was influenced by Hpi, LDi (*p* > 0.001, all), and modifications in the perfusion density in the SCP (*p* < 0.001) and DCP (*p* = 0.03) at the parafovea. In contrast, R1 + R2 RAD were associated with HbA1c (*p* = 0.02) and Hpi (*p* < 0.001). Conclusions: Microvascular changes and glucometabolic factors are key elements influencing the long-term morphofunctional alterations at the photoreceptor level in DM1.

## 1. Introduction

Type 1 diabetes mellitus (DM1) is an autoimmune disorder marked by the destruction of insulin-producing β-cells in the pancreatic islets, leading to chronic hyperglycemia and subsequent microvascular and neuronal complications. Among these complications, diabetic retinopathy (DR) stands as a significant cause of visual impairment and blindness in the working-age population worldwide [1].

The metabolic perturbations accompanying DM1 have been closely linked to the initiation and progression of DR. Hyperglycemia-driven oxidative stress, inflammation, and alterations in retinal microcirculation are accompanied by progressive structural and functional abnormalities of retinal neurons and glial cells [2,3].

Optical coherence tomography (OCT) allows for high-resolution imaging of retinal layers, revealing disruptions in the photoreceptor layer, a more accurate evaluation of macular edema, and the identification of several biomarkers of disease activity [4,5,6,7,8]. Recent developments in optical coherence tomography angiography (OCTA) have improved our knowledge of early microvascular changes in DR. This technology enables the precise anatomical correspondence of the different vascular networks, including the superficial (SCP), deep (DCP) capillary plexuses, and choriocapillaris (CC) [9,10,11,12,13,14]. These imaging modalities are critical in understanding microvascular changes in DR, such as capillary dropout, vascular remodeling, and perfusion deficits, which are closely associated with disease progression.

Along with traditional imaging techniques, which are now of routine use in clinical practice, more sophisticated methodologies have been employed to investigate the hypothesized link between microvascular damage and outer retinal abnormalities. These technologies, while not typically used in clinical settings, are more research-focused and designed to detect subtle structural and functional changes at the photoreceptor level [11,12,15,16]. Adaptive optics (AO) can offer an optimal lateral resolution (2–3 μm) capable of resolving single cells [17,18], revealing a direct relationship between the cone mosaic and microvascular perfusion in DM1 patients at early DR stages [19]. Further longitudinal analysis demonstrated DCP remodeling in response to the modifications in cone spacing, suggesting a compensatory mechanism of the DCP in supporting photoreceptors’ well-being. Moreover, cone spacing and arrangement exhibited significant modifications in response to SCP and CC perfusion [20]. In this intricate maze of microstructural modifications, retinal dysfunction can precede evident changes in the cone mosaic [21].

Multifocal electroretinography (mfERG) offers a spatially resolved assessment of retinal bioelectrical responses, allowing for a localized functional evaluation of the cone photoreceptors and bipolar cells [22]. This technique has been able to detect subtle functional alterations even before clinical manifestations of DR [23], making it a valuable tool for identifying early and subclinical functional neuroretinal changes [24]. Combining the functional insights of mfERG with the retinochoroidal information from OCT/OCTA and the microstructural resolution from AO provides a comprehensive view of the localized and early cellular changes that drive diabetic retinopathy progression.

This study aimed to explore the potential relationship between metabolic changes, structural derangements, and long-term functional alterations in DM1. By assessing OCT/OCTA, AO, and mfERG over a 4-year period, we aimed to identify the factors influencing photoreceptor viability over time, taking into account the metabolic changes. This in vivo investigation could pave the way for a better pathogenic understanding of the mechanisms leading to irreversible photoreceptor damage and possibly develop future strategies for preserving retinal function and arresting the advancement of DR.

## 2. Materials and Methods

### 2.1. Study Participants

A total of 22 DM1 patients (22 eyes) with mild nonproliferative diabetic retinopathy (NPDR) were included in this prospective observational evaluation. The study was conducted at IRCCS Bietti Foundation between September 2017 and May 2022. Baseline characteristics are summarized in Table 1.

The study complied with the tenets of the Declaration of Helsinki and received approval from the Institutional Review Board (IRB) of the IRCCS-Fondazione Bietti (RET03-22/12/2016). Written informed consent was obtained from all patients prior to inclusion in this study.

DM1 patients with a minimum age of 18 years were screened by experienced referral diabetologists (F.P. and S.F.) and enrolled only if they were under optimal glucometabolic control obtained through an insulin pump and without any concomitant systemic comorbidities. 

Patients were then evaluated by two experienced retinal specialists (M.P. and E.C.) through comprehensive ophthalmic evaluations and imaging, including color fundus photography, OCT, OCTA, and AO. The diagnosis of mild NPDR was based on the modified ETDRS severity scale [20] and was determined using both clinical examination and multimodal imaging at baseline. Exclusion criteria were as follows: the presence of other ophthalmic comorbidities, history of intraocular or vitreoretinal surgery, axial length > 26 mm and/or a spherical error exceeding 6 diopters, evidence of intraretinal cysts or macular edema, dioptric media opacities, and poor-quality images. Patients who progressed into a more advanced stage of DR or developed DMO were excluded from the analysis.

Ophthalmological examinations, multimodal imaging, and mfERG recordings were conducted at the baseline and subsequently on an annual basis for a maximum follow-up duration of 4 years.

### 2.2. Multimodal Imaging and Post-Processing

Color fundus photography (CFP) was captured using the Topcon TRC-50DX. Spectral-domain OCT was conducted using Heidelberg Spectralis OCT (HRA + OCT, Heidelberg Engineering, Heidelberg, Germany, software version 1.10.2.0) employing a minimum scanning protocol comprising a 20 × 20-degree square and 25 B-scans centered on the fovea. Swept-source OCTA was performed through PLEX Elite 9000 (software version: 1.7.027959). A 3 × 3 mm volume cube was acquired with eye-tracking and projection artifact removal tools. The different vascular slabs (SCP, DCP, and CC) were automatically segmented using the built-in software provided by PLEX Elite 9000. A single operator (S.F.) checked the segmentation boundaries for misalignment or segmentation errors and proceeded to a manual re-segmentation. A cut-off of 8/10 was arbitrarily chosen as the signal strength index (SSI) for optimal OCTA quality.

An AO retinal camera rtx1 (Imagine Eyes, Orsay, France) captured cone mosaic images, using an infrared illumination of 850 nm and a spatial resolution of 2 μm as previously described [19,20]. The images were montaged and aligned using the i2k Retinal Pro montage tool (DualAlign, Clifton Park, NY, USA). Cone cells were labeled and identified through an enhanced algorithm within the MATLAB Imaging Processing Toolbox (The Mathworks, Inc., Natick, MA, USA), using the Voronoi MATLAB function as previously described [19,25].

**Figure 1:**
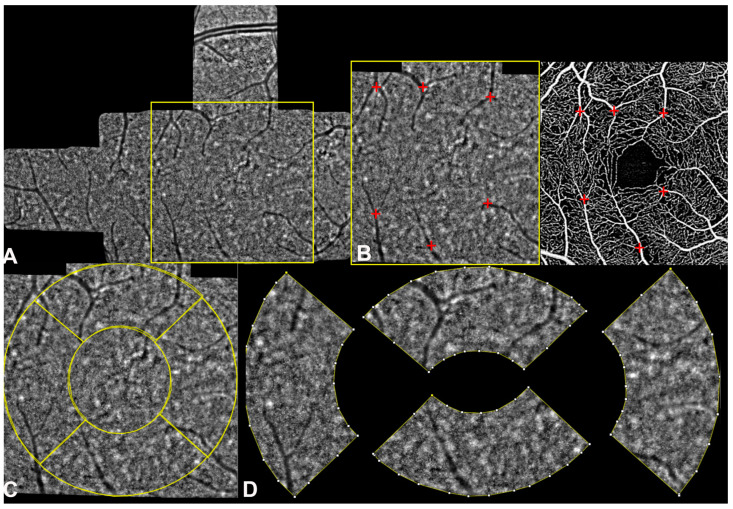
A schematization of the image post-processing: (**A**) adaptive optics (AO) montage was cropped into a 1.25 mm^2^ (square) using the open source software ImageJ (Fiji, https://imagej.net/Fiji/Downloads; software version 2.3.0/1.53f); (**B**) the cropped image was aligned and registered using the “landmark correspondence method” considering the superficial vessels seen on the superficial vascular slab (red crosses); (**C**) a customized ETDRS grid (outer circle of 1.25 mm, inner circle of 1 mm diameter) was overlaid to the registered images (yellow circle); (**D**) the grid comprised 4 distinct regions of interest (ROIs) representing the parafoveal sectors used for calculating AO metrics, including cone density (CD), linear dispersion index (LDi), and heterogeneity packing index (HPi).

OCTA images were post-processed using the open source software ImageJ (distributed by Fiji, NIH, Bethesda, MD, USA, https://imagej.net/Fiji/Downloads (accessed on 24 September 2024); software version 2.3.0/1.53f) for calculating the perfusion density (PD) and CC flow deficit percentage (FD%) with the previously described procedure^13^. The images were first aligned and registered using the “landmark correspondence method.” A customized ETDRS grid (outer circle of 1.25 mm, inner circle of 1 mm diameter) composed of 4 distinct ROIs representing the parafoveal sectors was overlaid to the registered images for quantitative sectorial analysis, as detailed previously [19,20]. For further details, see Figure 1.

For the purpose of the present study, the 4 parafoveal sectors were averaged, and the entire parafovea was used in the model to achieve the most reliable structure–function interpolation with the mfERG rings analyzed (see below).

The main outcome measures from AO included (a) cone density (CD)—number of cones per square millimeters (cones/mm^2^); (b) linear dispersion index (LDi)—distance between adjacent cones or cone spacing; (c) heterogeneity packing index (HPi%)—modifications in cones’ spatial distribution pattern or arrangement [25].

### 2.3. Multifocal Electroretinogram

The function of the foveal and parafoveal areas was explored using the VERIS mfERG system (VERIS Clinic TM version 4.9; Electro-Diagnostic Imaging, San Mateo, CA, USA) in accordance with the 2021 ISCEV standards [26]. The multifocal stimulus, composed of 61-scaled hexagons, was presented on a high-resolution, black-and-white monitor (size: 32 cm wide and 30 cm high) with a 75 Hz frame rate. The hexagonal pattern alternated between black (1 cd/m^2^) and white (200 cd/m^2^), covering 20° of the visual field. The monitor luminance was 100 cd/m^2^. The m-sequence had 2^13−1^ elements with a total recording time of 4 min divided into 8 segments. The fixation target was represented by a small red target in the center of the stimulation field, under continuous camera monitoring. The test was performed using focusing lenses if necessary.

MfERGs were performed under pharmacological mydriasis (1% tropicamide eye drops) and local anesthesia (0.4% benoxinate) to apply Dawson–Trick–Litzkow (DTL) contact electrodes on the lower eyelid. Ag/AgCl skin reference electrodes were applied on the corresponding outer canthi for binocular recordings, while a small Ag/AgCl skin electrode was positioned at the center of the forehead. As already published in our previous work [23], first-order kernel responses were analyzed after automatic artifact rejection, and the bioelectrical responses originating from concentric annular rings centered on the fovea were explored. Averaged responses were collected from five rings at progressively increasing distances from the fovea: (i) 0–2.5° (ring 1, R1); (ii) 2.5–5° (ring 2, R2); (iii) 5–10° (ring 3, R3); (iv) 10–15° (ring 4, R4); and (v) 15–20° (ring 5, R5).

**Figure 2:**
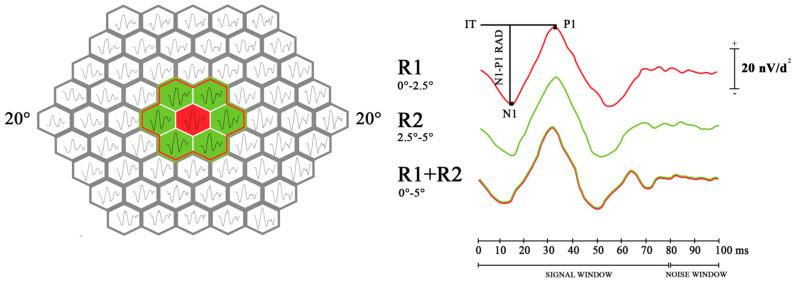
Multifocal electroretinogram (mfERG) of a representative case of a patient with type 1 diabeties; the mfERG layouts shown on the left panel include 61 averaged bioelectrical responses recorded in a single session. On the right panel, the resulting trace after first-order kernel measurements is presented: ring 1 (R1, 0–2.5° foveal eccentricity) is shown in red, ring 2 (R2, 2.5–5° foveal eccentricity) is displayed in green, and ring 1 + ring 2 (R1 + R2, 0–5° foveal eccentricity) are visible as superimposed red–green lines. N1–P1 response amplitude density (RAD, nV/deg^2^) and P1 implicit time (IT, milliseconds) are denoted by black arrows.

For the purpose of this study, to establish a potential topographical correlation between parameters describing morphological and functional changes in DM1 patients from almost superimposable areas, we selected the mfERG responses (P1 IT and N1-P1 RAD) derived from R1 (foveal area), R2 (parafoveal area) and from unified rings R1 + R2 (the total area from 0° to 5°, foveal + parafoveal area). The averaged responses were analyzed considering the peak-to-peak response amplitude density (RAD) measured in nanovolt/degree^2^ (nV/d^2^) from the first negative peak (N1) to the first positive peak (P1) of the waveforms, and the implicit time (IT) estimated on the first positive peak (P1) in milliseconds (msec) (Figure 2).

mfERG IT describes the time elapsed in the responses of the synapse between a photoreceptor (mainly macular cones) and the related bipolar cells, while mfERG RAD describes the amplitude of the bioelectrical response driven by photoreceptors–bipolar cells in a specific area, which measures the density of functioning cells in a specific area [22].

### 2.4. Statistical Analysis

Based on the results from our previous study [20], a sample size of 21 cases was estimated, with a significance level of 5% and a power of 80% (paired *t*-test, two tails). The Shapiro–Wilk test was used to verify the normality of distribution. Data are reported as mean ± standard deviation (SD). Pearson’s correlation coefficient was used to assess the relationship among parameters, considering a correlation coefficient with at least a moderate agreement (r > 0.4). A repeated-measure analysis of variance (ANOVA) was used to assess ERG parameter differences over time in the study group. The assumptions for repeated-measure ANOVA, including normality, sphericity, and independence, were evaluated. Mauchly’s test of sphericity was conducted to assess sphericity. If the assumption was violated (*p* < 0.05), Greenhouse–Geisser and Huynh–Feldt epsilon (ε) corrections were applied based on ε values (ε < 0.75: Greenhouse–Geisser; ε > 0.75 Huynh–Feldt). To overcome the possible limitations related to repeated-measure ANOVA, a linear mixed model analysis was performed. This approach allowed for handling missing data, provided a more robust analysis, and accounted for different covariance structures. Additionally, it offered the flexibility to treat repeated measures as either continuous or categorical variables, also dealing with small sample sizes [27]. Age, HbA1c, diabetes duration, CD, LDi, Hpi, SCP PD, DCP PD, and CC FD were considered fixed effects. The best-fitted model was chosen among the different models combining the mentioned features according to the Bayesian Information Criterion (BIC). BIC values were calculated for each model, with the lowest BIC indicating the best balance between goodness of fit and model parsimony. The criterion penalizes models with a higher number of parameters, helping to avoid overfitting. Models with substantially higher BIC values were considered less optimal and excluded. The final model was selected based on having the lowest BIC value, indicating that it provided the best fit for the data while maintaining interpretability to avoid unnecessary complexity. The *p*-value was set at 0.05 (two-sided) for all analyses. Statistical analysis was performed using SPSS software (IBM SPSS Statistics V.25) and RStudio software version 2022.07.1 (RStudio, PBC, Boston, MA, USA, URL http://www.rstudio.com/, accessed on 24 September 2024).

## 3. Results

The multifocal ERG parameters analyzed in the predictive model are summarized in Table 2.

**Figure 3:**
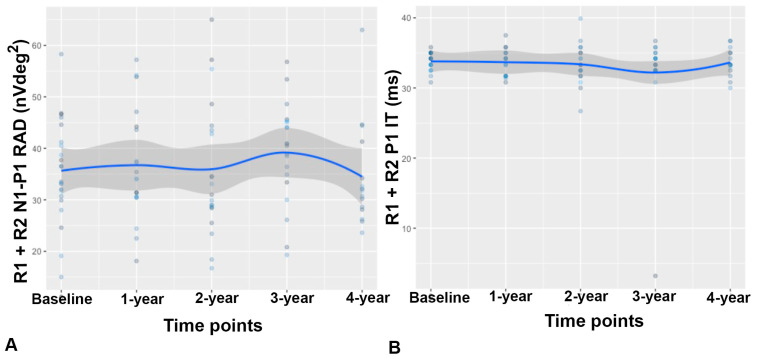
Longitudinal multifocal electroretinogram (mfERG) modifications. The spaghetti trend line graph shows the changes in mfERG responses: (**A**) unified rings R1 + R2 (total area from 0° to 5°, foveal + parafoveal area) response amplitude density (RAD) measured in nanovolt/degree^2^ (nV/deg^2^); (**B**) R1 + R2 implicit time (IT) measured in milliseconds (ms) of the first positive peak (P1).

No significant differences were noted over time in the averaged R1, R2, and R1 + R2 RAD and IT (*p* > 0.05, all). An almost stable trend line of the R1 + R2 averaged parameters, considering the loss of follow-up, is displayed in Figure 3.

**Figure 4:**
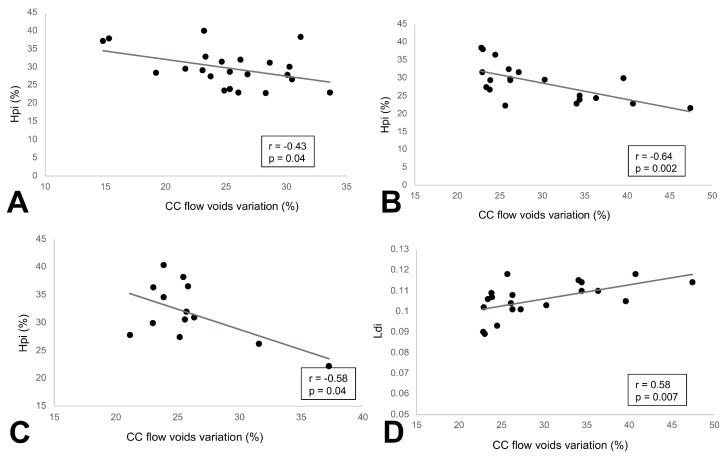
Scatterplots of the structural–microvascular correlations: (**A**) heterogeneity packing index (Hpi) was inversely correlated with choriocapillaris (CC) flow void variation (%) at baseline; (**B**) a similar correlation was found at 1-year follow-up and (**C**) 2-year follow-up; (**D**) linear dispersion index (LDi) exhibited a direct relationship with CC FD%.

**Figure 5:**
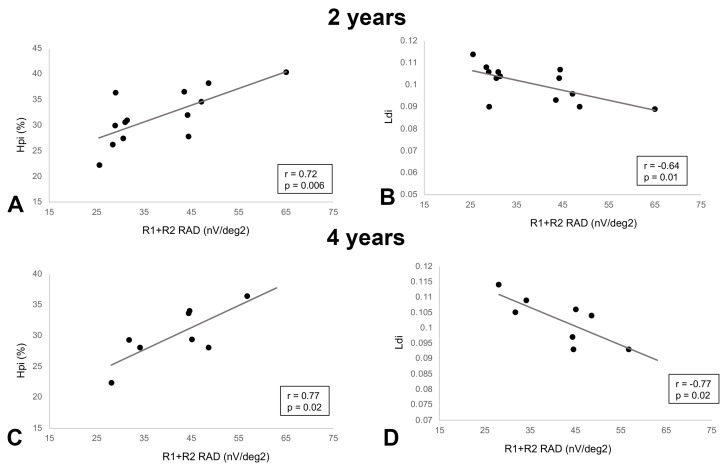
Scatterplots of the structural–functional correlations. At 2-year follow-up, (**A**) heterogeneity packing index (Hpi) demonstrated a direct association with R1 + R2 RAD (r = 0.72, *p* = 0.006); (**B**) linear dispersion index (LDi) showed an inverse relationship with R1 + R2 RAD (r = −0.64, *p* = 0.01). At 4-year follow-up, the same trend is appreciable for both (**C**) Hpi and R1 + R2 RAD (r = 0.77, *p* = 0.02) and (**D**) Ldi with R1 + R2 RAD (r = −0.77, *p* = 0.02).

At baseline, a history of longer diabetes duration was associated with decreasing R1 + R2 RAD values (r = −0.46, *p* = 0.03). Among the structural AO features, Hpi was inversely associated with CC FD at baseline (r = −0.43, *p* = 0.04), as well as 1-year (r = −0.64, *p*= 0.002), and 2-year (r = −0.58, *p* = 0.03) follow-ups, indicating that with increasing flow voids at the CC level, the Hpi tended to decrease in the first years of follow-up. An opposite trend was noted between Ldi and CC FD at 1-year follow-up (r = 0.58, *p* = 0.007) (Figure 4). Interestingly, a direct association between structural and functional parameters was noted starting from the second year of follow-up. At year 2, a decreased Hpi was positively correlated with a decrease in the R1 + R2 RAD (r = 0.72, *p* = 0.006). The same trend was noted in year 4 (r = 0.77, *p* = 0.02). Similarly, LDi was negatively associated with R1 + R2 RAD (r = −0.64, *p* = 0.01) at 2-year and 4-year (r = −0.77, *p* = 0.02) follow-ups (Figure 5).

### Factors Influencing the Functional Parameters Evaluated Through mfERG

The best-fitted model according to BIC values included a combination of the duration of diabetes, HbA1c level, CD, Hpi, LDi, SCP, DCP, and CC at the parafovea location on the functional parameters. Table 3 reports the main effects and the *p*-values for the parameters in the model.

When considering R1, the only factor influencing the IT was HbA1c, while R1 RAD was affected by Hpi and CC FD%. R2 IT was significantly influenced by age and the RAD by Hpi. The analysis of R1 + R2 revealed more interesting associations. The main factors influencing the R1 + R2 IT included a combination of cone metrics on parafoveal location, Hpi, and LDi, but also microvascular modifications in the parafovea at the level of SCP and DCP. The only factors affecting R1 + R2 RAD were HbA1c and Hpi.

## 4. Discussion

**Figure 6:**
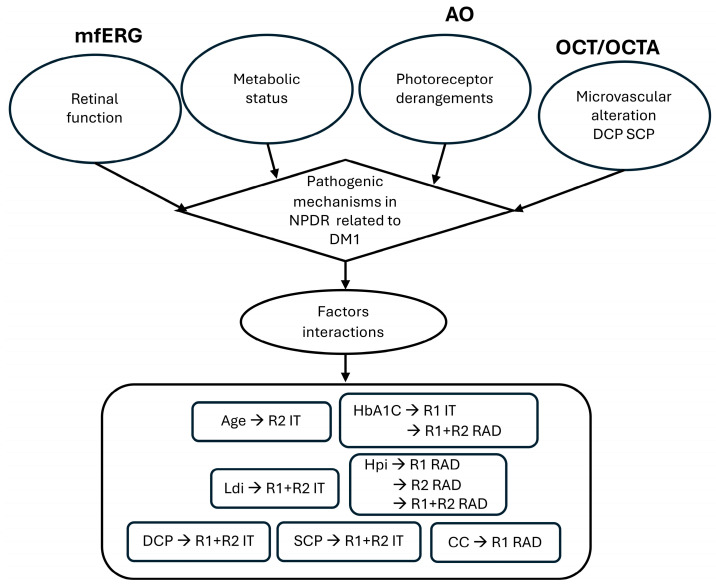
A block algorithm summarizing the presented solution. mfERG: multifocal electroretinogram; AO: adaptive optics; OCT/OCTA: optical coherence tomography/optical coherence tomography angiography; RAD: response amplitude density measured in nanovolt/degree2 (nV/d^2^); IT: implicit time in milliseconds (ms). R1: from 0° to 2.5°; R2: from 2.5° to 5°; R1 + R2 unified rings R1 + R2: total area from 0° to 5° (foveal + parafoveal area).

This study further refines our previous DM1 model [19], considering metabolic, microvascular, and photoreceptor modifications over 4 years in mild NPDR. The model integrates mfERG functional evaluations with these parameters to understand factors interfering with photoreceptor dysfunction. An inference algorithm summarizing the presented work is reported in Figure 6.

The population enrolled involved patients with DM1 without systemic comorbidities at an initial stage of NPDR maintained over a 4-year period. Therefore, a relative stability of the functional parameters over time may be conceivable. Despite this, some interesting relationships were evident between functional, metabolic, and structural features. Notably, longer diabetes duration at baseline was the main factor correlated with a reduction in R1 + R2 amplitude. This further corroborates our previous findings, showing that retinal dysfunction significantly correlates with age and diabetes duration, which indicates that functional retinal abnormalities are related to the onset of diabetes [23]. Previous predictive models of DR development have identified a high predictive rate of new retinopathy according to the mfERG implicit time delays [28,29,30]. The application of mfERG has unveiled significant retinal dysfunction before the development of a clinically evident retinopathy [29] and a precocious neuronal signaling impairment involving outer and middle retinal layers, depending on diabetes more than on DR [23].

In our model, integrating AO into retinal imaging enabled the high-resolution imaging of individual photoreceptor cells, surpassing the limitations of traditional imaging techniques. In this regard, a direct linear relationship between structural and functional parameters was evident in the second year of follow-up, where decreased photoreceptor packing (Hpi) correlated with reduced foveal and parafoveal photoreceptors and bipolar cell function (R1 + R2 RAD). This trend persisted at the last available follow-up (year 4). Similarly, increased cone spacing (Ldi) at years 2 and 4 was related to reduced R1 + R2 amplitudes. These findings suggest a direct structure–function relationship at almost superimposable retinal locations that remains evident as the disease progresses, leading to progressive photoreceptor rearrangement and localized retinal dysfunction, which may not be apparent in a global functional evaluation.

The predictive models added crucial insights to the present study. Using ring analysis, we found that modifications to the foveal function (R1 RAD) depended on both Hpi and CC FD%, while HbA1c was the only factor influencing implicit time. Additionally, parafoveal function (R2 RAD) was significantly influenced by Hpi, whereas R2 IT was highly dependent on age.

Moreover, the retinal function in the foveal + parafoveal areas (R1 + R2 RAD) was influenced by both HbA1c at baseline and Hpi rearrangement, whereas response timing delay (R1 + R2 IT) presented significant associations with both Hpi and LDi and microvascular parameters, including SCP and DCP PD%. mfERG implicit times have been shown to correlate with the severity of retinopathy, making it a highly sensitive method for assessing local retinal function in the early stages [31]. In this context, retinal hypoxia is considered the primary factor responsible for implicit time delays [31,32,33]. Our findings may further support the hypothesis of hypoxia-related photoreceptor dysfunction, as we observed an association between microvascular perfusion and implicit time delays in both the foveal and parafoveal regions.

Perfusion changes in the DCP were found to manifest in the early stages of DR, occurring even before the onset of clinically detectable microvascular pathological alterations [14,34,35,36]. Such perfusion changes, more readily observed in the SCP, were characterized by an initial compensatory vasodilation in response to the hypoxia, followed by a gradual reduction in perfusion over the course of years [13,20,37]. This compensatory mechanism serves to increase the blood transit into the tissue, likely in response to ischemic changes occurring within the middle and DCP layers, thereby preserving adequate perfusion, limiting microvascular damage, and impeding disease progression [13,37,38,39,40]. Based on all these considerations, we could hypothesize that after 2 years of FU, the observed loss of autoregulation of the SCP and DCP may influence PRs rearrangements and function. Notably, DCP and CC impairments were both found to correlate with areas of reduced retinal sensitivities determined by microperimetry [21,41]. A plausible timeline of early microvascular and structural modifications is summarized in Appendix A.

It is also important to highlight that functional abnormalities have also been detected in individuals with diabetes without DR [42]. A possible explanation for this finding resides in an early or subclinical choriocapillaris impairment [33,43,44,45]. Histopathological findings have revealed choriocapillaris involvement in diabetic eyes, with basement membrane thickening leading to capillaries narrowing, leading to ischemia that can affect the outer retina [15,46,47,48]. Abnormalities of CC perfusion in diabetes have also been confirmed in vivo by using OCTA, demonstrating a direct relationship between CC flow deficits and DR severity [15,49,50,51]. Our mfERG findings possibly support a direct contribution of choroidopathy in diabetic photoreceptor degeneration, revealing that CC changes seem to predominantly affect the R1 amplitude and, thus, the foveal region. This regional predilection may be attributed to the early impairment of CC in DR. The foveola, which contains the highest density of cone photoreceptors, relies exclusively on choroidal perfusion for its metabolic needs. As the CC begins to deteriorate in the early stages of DR, the foveola’s photoreceptors become particularly vulnerable to ischemic damage due to the lack of alternative blood supply from the retinal vasculature [51,52,53]. In our previous study [54], we also observed a correlation that approached significance between R1RAD and CC FD%, lending support to the hypothesis of a probable association between CC vascular dropout and photoreceptors’ dysfunction as an early pathophysiological retinal change in DM1.

Also, it is noteworthy to underline that in NPDR-examined eyes, cone packing (Hpi) was consistently associated with retinal dysfunction, with RAD significantly reduced in R1, R2, and even more in R1 + R2, and IT was significantly delayed in R1 + R2. Additionally, cone spacing (LDi) was correlated with R1 + R2 IT, indicating that structural–functional impairment affects both the foveal and the parafoveal area. In contrast, microvascular modifications (particularly in the SCP and DCP) influence retinal function only when considering diffuse areas, enclosing the whole fovea and parafovea. This suggests that localized structural and neurofunctional signaling alterations in the outer and middle retina are affected by diffuse retinal vascular disturbances. In this context, CC changes do not interfere with the parafoveal function but affect the fovea, as demonstrated by the correlation between CC FD% and R1 RAD. In our previous findings [54], we identified a trend of a significant correlation (*p* = 0.067) between R1 RAD and CC FD% in a similar cohort of NPDR DM1 eyes. This difference may be ascribed to the extremely different follow-up of NPDR disease, as the previous study was a cross-sectional observation [40].

The strengths of the present study include a long follow-up enrolling DM1 patients without systemic comorbidities that may bias the results observed, as well as the creation of a predictive model including a wide range of factors that can play a role in the disease pathogenesis. Despite these strengths, this study suffers from several limitations that deserve careful consideration. Such limitations include missing data, the loss of follow-up, and the lack of a control group, as well as the simultaneous acquisition of multiple imaging modalities, which can affect patients’ cooperation and imaging acquisition but can also be time-consuming in a clinical setting.

In conclusion, this study explores the complex interplay between microvascular, metabolic, and structural changes in the retina and their long-term functional implications in DM1 patients with mild nonproliferative diabetic retinopathy. By integrating multimodal conventional imaging techniques such as OCT/OCTA with AO and mfERG, we hypothesized that changes in microvascular perfusion and photoreceptor arrangement are closely associated with retinal function. Structural photoreceptor changes (Hpi and LDi) were significantly correlated with mfERG response amplitude and implicit time, suggesting that cone packing and spacing influence functional retinal changes. Microvascular alterations in the SCP and DCP contributed to retinal dysfunction, particularly affecting response timing, while perfusion changes in the CC were associated with foveal dysfunction. Based on our findings and the previous literature, we hypothesized that the involvement of the CC plays a pivotal role in the disease progression, influencing the structural reorganization of the retina from the initial stages of DR. As the disease progresses, the microvascular integrity of both SCP and DCP, along with effective glucometabolic control, seem to emerge as critical determinants shaping the long-term structural and functional modifications at the level of photoreceptors. Our study further highlights the importance of adequate metabolic control as the Hb1Ac levels have demonstrated a crucial role in influencing photoreceptor function. Future research should focus on larger cohorts and longer follow-up periods to validate these findings and explore potential therapeutic interventions that could preserve retinal function and prevent progression to advanced diabetic retinopathy.

## Figures and Tables

**Table 1:** Demographic baseline characteristics of non-proliferative diabetic retinopathy (NPDR) patients enrolled.

Variables	NPDR (n = 22)
Age (years)	42.6 ± 10.1
Gender (female,%)	13 (59.1%)
Duration of diabetes (years)	19.6 ± 9.3
HBA1c (%)	7.3 ± 0.8

Quantitative data are presented as mean ± standard deviation (SD).

**Table 2:** Multifocal electroretinogram parameters at the different time points.

	R1		R2		R1 + R2	
	IT	RAD	IT	RAD	IT	RAD
	(ms)	(nV/deg^2^)	(ms)	(nV/deg^2^)	(ms)	(nV/deg^2^)
Baseline	34.6 ± 4.2	79.4 ± 20.1	33.2 ± 2.7	34.5 ± 8.3	33.8 ± 1.3	35.6 ± 9.7
1-year	34 ± 3.9	83.1 ± 19	33.5 ± 2.3	36.4 ± 10.9	33.7 ± 1.8	36.7 ± 10.8
2-year	34.2 ± 3.3	75.7 ± 29.8	33.5 ± 2.9	34.1 ± 12.3	33.4 ± 2.7	35.9 ± 13.1
3-year	34.2 ± 1.8	86.8 ± 25.3	33.4 ± 1.7	37.1 ± 9.7	32.3 ± 6.8	39.2 ± 9.8
4-year	33.9 ± 4.1	87.6 ± 34.4	33.3 ± 2.5	34.9 ± 11.1	33.6 ± 1.97	34.5 ± 10.2
*p*-value *	0.98	0.70	0.05	0.88	0.64	0.75

Data are expressed as mean ± standard deviation. * *p*-value was estimated through repeated-measure analysis of variance (ANOVA) comparing the mfERG parameters (IT, RAD in R1, R2, and R1 + R2) ∗ time. RAD: response amplitude density (nanoVolt/degree^2^), IT: implicit time (milliseconds).

**Table 3:** *p*-values for the fixed effects of the best model according to the BIC values.

	R1		R2		R1 + R2	
	RAD(nV/deg^2^)	IT(ms)	RAD(nV/deg^2^)	IT(ms)	RAD(nV/deg^2^)	IT(ms)
Age	-	0.15	-	<0.001 *	-	-
Diabetes duration	-	0.26	0.78	0.20	-	0.17
HbA1c	-	0.03 *	0.08	0.75	0.02 *	0.19
CD	0.43	-	-	-	0.62	-
Hpi	0.02 *	-	0.009 *	-	<0.001 *	<0.001 *
LDi	-	-	0.10	0.93	0.08	<0.001 *
SCP	0.43	0.72	-	0.74	0.53	<0.001 *
DCP	0.12	-	0.29	0.96	0.14	0.03 *
CC	0.01 *	-	0.24	-	0.14	0.37

HbA1c: glycosylated hemoglobin; CD: cone density, Hpi: heterogeneity packing index, LDi: linear dispersion index; SCP: superior capillary plexus perfusion density; DCP: deep capillary plexus perfusion density; CC: choriocapillaris flow voids (%). Variables not included in the models according to BIC are labeled as ‘-’. * Significant *p*-values (*p* < 0.05).

**Figure A1:**
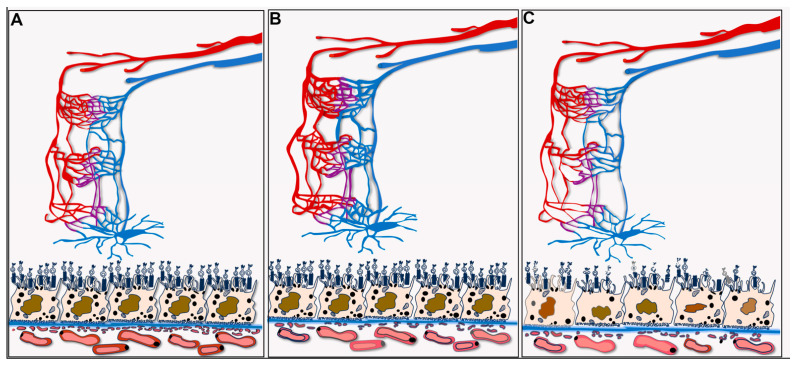
The hypothesized early microvascular and structural modifications that occur in patients with type 1 diabetes mellitus with mild nonproliferative diabetic retinopathy: (**A**) normal condition; for representative purposes, only microvascular plexuses, including superficial vascular plexus and intermediate and deep vascular plexuses, are depicted. Structural modifications are highlighted in the outer retina, including photoreceptor cells and retinal pigment epithelium with underlying Bruch’s membrane (blue). Choriocapillaris and choroidal vasculature are also presented; (**B**) initial compensatory vasodilation occupies mainly the superficial vascular plexus, while a progressive choriocapillaris and choroidal impairment are also observed; (**C**) loss of microvascular autoregulation, superficial vascular plexus returned to basal perfusion, but the deep vascular plexus is significantly attenuated. A choriocapillaris impairment is also significant at this stage, accompanied by an initial photoreceptor disorganization and dysfunction.

## Data Availability

The data are available upon reasonable request to the corresponding author due to privacy restrictions.

## Nomenclature

OCT	Optical coherence tomography
OCTA	Optical coherence tomography angiography
mfERG	Multifocal electroretinogram
AO	Adaptive optics
CD	Cone density
LDi	Linear dispersion index
HPi	Heterogeneity packing index
DR	Diabetic retinopathy
SCP	Superficial capillary plexus
DCP	Deep capillary plexus
CC	Choriocapillaris
HbA1c	Hemoglobin A1c (glycated hemoglobin)
NPDR	Nonproliferative diabetic retinopathy
DM1	Type 1 diabetes mellitus
ETDRS	Early Treatment Diabetic Retinopathy Study
CFP	Color fundus photography
RAD	Response amplitude density
IT	Implicit time
